# Carcinome épidermoide conjonctival négligé à propos d'un cas

**DOI:** 10.11604/pamj.2014.17.226.3561

**Published:** 2014-03-24

**Authors:** Hakima Elouarradi, Moulay Zahid Bencherif

**Affiliations:** 1 Université Mohammed V Souissi, Service d'Ophtalmologie A de l'Hôpital des Spécialités, Centre Hospitalier Universitaire, Rabat, Maroc

**Keywords:** Carcinome épidermoide, conjonctive, œil, squamous carcinoma, conjunctiva, eye

## Image en medicine

Une femme, âgée de 70 ans, monophtalme de l'oeil droit (oeil gauche en phtyse, perdu suite à un traumatisme oculaire à l'enfance), adressée pour une volumineuse tumeur infiltrant le globe oculaire droit (A). L'interrogatoire révèle l'existence d'une tumeur conjonctivale nasale limbique négligée depuis quatre ans. L’étude anatomopathologique de la biopsie confirme le diagnostic de carcinome épidermoide conjonctival bien différencié dyskératosique. Le scanner orbitocérébral montre une masse tumorale infiltrant le globe oculaire, se réhaussant discrètement après injection du produit de contraste (B) avec effraction osseuse du toit et du plancher de l'orbite homolatéral. Le bilan d'extension était négatif. On a pratiqué une exentération (C, D). Le carcinome épidermoide conjonctival est une tumeur rare. L'exposition solaire prolongée est unanimement reconnue comme un facteur prédisposant. Il concerne le plus souvent des personnes âgées, sans prédominance de sexe. Quand il survient à des âges plus précoces, une infection par le VIH doit être recherchée. L'extension du CEC se fait avant tout localement par continuité en surface, et en profondeur rendant l'exérèse chirurgicale complète parfois difficile, d'où une exentération s'impose dans ces cas ainsi qu'un curage ganglionnaire. Devant toute tumeur conjonctivale suspecte de part son aspect, sa symptomatologie douloureuse, ou encore sa croissance brutale, il faut procéder à une exérèse chirurgicale soigneuse. L’étude anatomopathologique de la pièce est la seule à garantir le diagnostic de certitude. Il reste à sensibiliser la population aux facteurs de risque et à l'importance de la protection contre les rayons ultraviolets.

**Figure 1:**
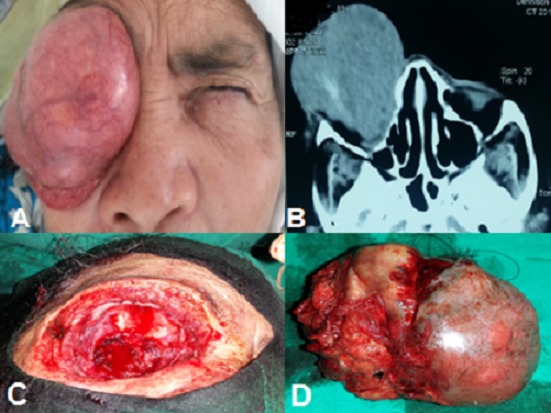
A) Grosse tumeur infiltrant le globe oculaire droit; B)Scanner orbitaire objectivant une volumineuse masse occupant l'orbite droite; C) Aspect per opératoire de l'exentération réalisée; D) Aspect de la pièce opératoire

